# NOXA expression is downregulated in human breast cancer undergoing incomplete pathological response and senescence after neoadjuvant chemotherapy

**DOI:** 10.1038/s41598-023-42994-2

**Published:** 2023-09-23

**Authors:** Sofian Al Shboul, Mohammed El-Sadoni, Ahmad Alhesa, Nisreen Abu Shahin, Dua Abuquteish, Ola Abu Al Karsaneh, Elham Alsharaiah, Mohammad A. Ismail, Liliya Tyutyunyk-Massey, Moureq R. Alotaibi, Victoria Neely, Hisashi Harada, Tareq Saleh

**Affiliations:** 1 Department of Pharmacology and Public Health, Faculty of Medicine, The Hashemite University, Zarqa, 13133 Jordan; 2 Department of Pathology, Microbiology and Forensic Medicine, School of Medicine, The University of Jordan, Amman, 11942 Jordan; 3 Department of Microbiology, Pathology and Forensic Medicine, Faculty of Medicine, The Hashemite University, Zarqa, 13133 Jordan; 4 Department of Pathology, King Hussein Medical Center, Royal Medical Service, Amman, 11942 Jordan; 5 Cell Therapy Center, The University of Jordan, Amman, 11942 Jordan; 6 Frederick National Laboratory for Cancer Research, Frederick, MD USA; 7 Department of Pharmacology and Toxicology, College of Pharmacy, King Saud University, Riyadh, Saudi Arabia; 8 Philips Institute for Oral Health Research, School of Dentistry, Massey Cancer Center, Virginia Commonwealth University, Richmond, VA USA

**Keywords:** Breast cancer, Cancer therapy, Tumour biomarkers, Tumour-suppressor proteins, Cell-cycle exit

## Abstract

Neoadjuvant chemotherapy (NAC) is a frequently utilized approach to treat locally advanced breast cancer, but, unfortunately, a subset of tumors fails to undergo complete pathological response. Apoptosis and therapy-induced senescence (TIS) are both cell stress mechanisms but their exact role in mediating the pathological response to NAC is not fully elucidated. We investigated the change in expression of *PAMIP1*, the gene encoding for the pro-apoptotic protein, NOXA, following NAC in two breast cancer gene datasets, and the change in NOXA protein expression in response to NAC in 55 matched patient samples (pre- and post-NAC). *PAMIP1* expression significantly declined in post-NAC in the two sets, and in our cohort, 75% of the samples exhibited a downregulation in NOXA post-NAC. Matched samples that showed a decline in NOXA post-NAC were examined for TIS based on a signature of downregulated expression of Lamin-B1 and Ki-67 and increased p16^INK4a^, and the majority exhibited a decrease in Lamin B1 (66%) and Ki-67 (80%), and increased p16^INK4a^ (49%). Since our cohort consisted of patients that did not develop complete pathological response, such findings have clinical implications on the role of TIS and NOXA downregulation in mediating suboptimal responses to the currently established NAC.

## Introduction

Breast cancer is the most common malignancy in women, accounting for more than 31% of newly diagnosed female cancers, and is still responsible for around 60% of all cancer-related deaths^1^. Surgical resection remains the cornerstone of breast cancer management^2^, frequently complemented with conventional chemotherapy either as adjuvant or neoadjuvant chemotherapy (NAC)^3^. Locally advanced breast cancer is often managed with NAC prior to surgical therapy, which is directed at reducing tumor size, facilitating breast conservation surgery, and improving survival rates^4^. The response to NAC is usually assessed postoperatively based on the eradication of pathologically identifiable breast tumor cells (ypT0 ypN0 or ypT0/is ypN0), which is often referred to as complete pathological response (pCR)^5^. Importantly, patients who develop partial or incomplete pathological response to NAC have lower survival outcomes^6^. Therefore, a deeper understanding of the molecular processes that develop in response to NAC is required to predict treatment efficacy. Apoptosis and cellular senescence are two established cell stress responses to agents used as NAC in preclinical models^7, 8^; nevertheless, there is a limited understanding of their role in clinical cancer in response to NAC.

Apoptosis is a system of programmed cell death that is often induced in tumor cells in response to therapy-induced DNA damage, oxidative stress and suborganellar damage^9^. Cells undergo apoptosis through two main pathways that involve the activation of several caspases, namely, the extrinsic (death receptor) pathway and the intrinsic (mitochondrial) pathway^10^. The intrinsic pathway is triggered by events that increase the mitochondrial outer membrane permeabilization (MOMP) leading to the release of cytochrome c, caspase activation and execution of apoptosis^11^. Members of the BCL-2 protein family play a crucial role in regulating the MOMP and can exert pro-apoptotic and anti-apoptotic activity through BH3 domain-mediated interactions^12^. Pro-apoptotic BCL-2 family members include BAX, BAK, BAD, BID, BIM, PUMA, NOXA, and others^13^. NOXA, a BH3-only protein, plays a critical role in inducing tumor cell apoptosis^14^, primarily in response to DNA damaging, cytotoxic anticancer drugs^15^. NOXA facilitates apoptosis indirectly through binding the anti-apoptotic protein, MCL-1 and interfering with its ability to restrain BAK-mediated increase in the MOMP^16^. Accordingly, low NOXA expression has been described to contribute to apoptosis resistance in tumor cells^17–19^, and poor survival outcomes in breast cancer patients^20^. Therefore, measuring the expression levels of NOXA protein and mRNA of its coding gene, *PAMIP1*, in breast cancer tissues could reflect, in part, the status of apoptosis induction in response to NAC.

Therapy-Induced Senescence (TIS) has emerged as a fundamental mode of tumor cell stress responses^21^. We and others have recently demonstrated that TIS represents a component of the breast cancer response to NAC especially in tumors that undergo partial pathological response^7, 22–24^. Accumulating evidence suggests that TIS mediates beneficial short-term outcomes in cancer therapy but can have more deleterious effects in the long-term^25^. As such, the use of senolytics to eliminate senescent tumor cells has been proposed as a novel cancer treatment^26, 27^. Interestingly, the report by Shahbandi et al. demonstrated that a subset of chemotherapy-induced senescent breast tumor cells is resistant to senolysis by BH3 mimetics which was conferred by low NOXA expression^28^. Thus, it is largely unknown whether low NOXA expression is associated with the development of TIS in response to NAC in breast cancer.

In this work, we utilized publicly available gene sets of breast cancer patients that were exposed to NAC to explore the overall change of *PAMIP1* gene expression before and following NAC. We further performed immunostaining to investigate the change in NOXA protein expression levels in response to NAC in a cohort of breast cancer patients whose matched samples were collected pre- and post-NAC. We also examined the relationship between the expression of NOXA protein in tumor samples with potential evidence of TIS.

## Methods

### Patients’ sample

In this work, we utilized a sample of 55 breast cancer patients of stages I-III collected from the Department of Pathology at Jordan Royal Medical Services (JRMS) (n = 55). For each patient, we collected the core-needle biopsy sample (obtained before the exposure to any type of NAC) used originally for diagnostic purposes and the post-mastectomy tumor sample (after the completion of NAC), both archived as formalin-fixed paraffin-embedded (FFPE) sample blocks. Subsequently, matched pre-NAC and post-NAC tumor tissues for each patient were prepared for histochemical analysis. Tumor tissue samples and patients’ data were obtained based on protocols approved by the Institutional Review Board committees at JRMS (6/2021), the Hashemite University (No. 3/5/2018/2019), and The University of Jordan (No. 237/2021) in accordance with the ethical standards as laid down in the 1964 Declaration of Helsinki and its later amendments. Obtaining informed consents for this work was waived by both IRB protocols since all the samples used for this study were surplus (archived) tumor tissue samples, and that patients undergoing surgery or biopsy collection provide informed consent to donate any excess tissue (i.e., beyond that needed for clinical purposes).

The inclusion criteria for selecting the sample were: (1) age between 18 and 90 years; (2) an established diagnosis of one type of breast cancer including invasive ductal carcinoma (IDC) and invasive lobular carcinoma (ILC); (3) receiving one type of NAC prior to undergoing surgical resection; and (4) developing partial or poor pathological response to NAC. On the other hand, the exclusion criteria were as follows: (1) unavailability of FFPE tumor samples pre- and post-NAC; (2) breast cancer patients diagnosed with ductal carcinoma in situ (DCIS); (3) patients who received any kind of hormonal treatment. The main clinicopathological characteristics of the 55 breast cancer patients included in this study are provided in Table 1. The average age of patients in the sample was 49 years (range 28–70 years old).

**Table 1:** Clinicopathological characteristics of the patients’ sample.

	Matched breast cancer samples (n = 55)	
Breast cancer subtype	IDC	48 (87%)
	ILC	3 (6%)
	Other	4 (7%)
Stage	Stage I	25 (45%)
	Stage II	16 (29%)
	Stage III	14 (26%)
Grade	Grade 1	1 (2%)
	Grade 2	25 (45%)
	Grade 3	29 (53%)
Lymphovascular invasion	Present	27 (49%)
	Not identified	28 (51%)
Lymph node involvement	Positive	34 (62%)
	Negative	21 (38%)
Luminal A	ER + /PR + /HER2 -	34 (64%)
Luminal B	ER + /PR + /HER2 +	15 (27%)
HER2 +	ER-/PR- & HER +	4 (7%)
TNBC	ER-/PR-/HER2-	2 (4%)
ER	Positive	44 (80%)
	Negative	11 (20%)
PR	Positive	37 (67%)
	Negative	18 (33%)
HER2	Positive	19 (34%)
	Negative	36 (66%)

IDC, Invasive ductal carcinoma; ILC, Invasive lobular carcinoma; ER, Estrogen receptor; PR, Progesterone receptor; HER2, Human epidermal growth factor receptor 2; TNBC, Triple-negative breast cancer.

### Immunohistochemistry (IHC) and immunofluorescent staining

Sections of 4 µm thickness were obtained from FFPE breast cancer tissue blocks using a manual microtome (LEICA RM2235, Manual Rotary Microtome) and were placed on positively charged slides. Slides were dried at 65°C for 30 min to melt the paraffin wax and then placed in fresh xylene two times, 5 min each followed by rehydration in descending ethanol series (100%, 95%, and 70% ethanol; 3 min each) before being rinsed in deionized water. Antigen retrieval was achieved by placing the slides in jars containing sodium citrate buffer (pH 6.0) for Lamin B1 and p16^INK4a^, Tris–EDTA buffer (pH 9.0) for Ki-67 in a water bath at 95*˚*C for 60 min. Antigen retrieval for NOXA was carried out in a water bath at 95 °C in sodium citrate buffer (pH 6.0) for 20 min. Slides were then left to cool down at room temperature for 20 min and were washed twice with phosphate-buffered saline (PBS) containing 0.2% Tween 20 (CAS 9005-64-5, Sigma Aldrich, St. Louis, USA). Subsequently, slides were treated with 3% hydrogen peroxide in PBS to suppress endogenous peroxidase activity before being washed in PBS. Blocking was performed using 5% bovine serum albumin (Atlas Medical, Germany) for 60 min at room temperature. Next, sections were incubated with the following mouse monoclonal primary antibodies: anti-Lamin B1 (1:600, clone 4001, Cat. No. NBP2-59,783, Novus biologicals, USA), anti-Ki-67 (1:600, ab279653, Lot number: GR3397415-10, Abcam, Cambridge, United Kingdom), anti-p16^INK4a^ (1:250, clone 1D7D2A1, Cat. No. NBP2-37736, Novus biologicals, USA) and anti-NOXA (1:400, clone 114C307.1, Cat. No. NB600-1159, Novus biologicals, USA) for 2 h at room temperature. Slides were then rinsed in PBS and incubated with the super enhancer reagent (Super Sensitive™ Polymer-HRP IHC Detection, QD440-XAKE, BioGenex, USA) for 25 min at room temperature for Poly-HRP enhancement before being washed with PBS. Next, slides were incubated with HRP-Polymer anti-Mouse/Rabbit reagent (Super Sensitive™ Polymer-HRP IHC Detection, QD440-XAKE, BioGenex, USA) for 30 min at room temperature. Visualization of the immunostaining was achieved with 3, 3′-diaminobenzidine tetrahydrochloride (DAB) for 8 min. Slides were counterstained with Mayer's hematoxylin solution and treated with lithium carbonate solution for 30 s. Finally, slides were dehydrated with an ascending series of ethanol (70%, 95%, and 100% ethanol; 3 min each), cleared with fresh xylene, and cover slipped with dibutyl phthalate in xylene (DPX) mounting media (#BCBX0183, Sigma, Germany).

### NOXA protein expression scoring

The diagnosis of invasive breast carcinoma was established at The Pathology Department at JRMS. Two independent pathologists (authors DQ and OAAK) performed pathological evaluation of the breast tissue samples and the immunohistochemical scoring of NOXA protein expression using a light microscope (Labo America, USA). Immunostaining was assessed for core-needle biopsies (pre-NAC) and post-mastectomy tumor section (post-NAC) simultaneously for nuclear and cytoplasmic staining. Both staining patterns were observed in combination in many tumor cells, while others harbored one staining pattern—nuclear or cytoplasmic. Any degree of intensity (weak, moderate, or strong) was considered positive for NOXA expression. Eyeballing of the percentage of positive staining in tumor cells was performed by the two pathologists with a consensus reached for each percentage. Numbers were adjusted and rounded to the nearest tenth. The complete absence of staining was considered negative and scored zero.

### Senescence-associated proteins expression scoring

The signature of the induction of Therapy-Induced Senescence (TIS) in our matched breast cancer cohort was determined by comparing the change in the protein expression (pre-NAC vs. post-NAC) of three senescence-associated biomarkers, namely, Lamin B1, Ki-67 and p16^INK4a^^24^. Senescence-associated biomarkers were scored based on the median percentage of DAB-stained tumor cells for each marker by two independent pathologists (authors NAS and EA) using light microscopy (Olympus Optical, Tokyo, Japan). Scoring was performed semi-quantitatively in the area of most prominent staining of the tissue slides measuring stained cells and/or area ratio. For any given matched set, a decrease in Lamin B1 and Ki-67 expression, and a concomitant increase in p16^INK4a^ expression following the exposure to NAC was considered positive for TIS. All the other possibilities were considered negative or inconclusive for TIS. The inability to establish TIS based on the three markers utilized in this study and the proposed senescence signature do not rule out definitive induction of senescence in the examined samples.

### Assessment of PAMIP1 gene expression

Two gene expression data sets were accessed as normalized and processed RNA-seq data from the GEO database^29^. The first gene set (GSE28844: https://doi.org/10.1371/journal.pone.0053983) contained data from 33 breast cancer patients of which one sample was collected from pre-NAC only, four samples were obtained post-NAC only, and the remaining 28 were paired samples (pre- and post- NAC) from breast cancer patients that received anthracycline and taxane-based chemotherapy^30^. The second gene set (GSE21974: https://doi.org/10.3892/or.2011.1392) included 32 breast tumor patients of which there were seven samples collected only from the pre-NAC patients whilst 25 patients contributed with samples that were collected before and after receiving epirubicine and cyclophosphamide followed by docetaxel^31^. In both datasets, the pre-NAC tumor samples were collected using core-needle biopsy while post-NAC tumor samples were obtained through surgical resection^30, 31^.

### Subcellular fractionation and western blotting

MDA-MB-231 cells were seeded at 2.0 × 10^6^ in 10 cm dishes with DMEM (Thermo Fisher #10569010) supplemented with 10% heat-inactivated fetal bovine serum (FBS) and 100 μg/ml penicillin G/streptomycin at 37 °C in a humidified, 5% CO_2_ incubator. Cells were treated with 0 (DMSO), 0.5 and 1 µM doxorubicin (Dox) for 48 h. Subcellular fractionation was conducted using the Cell Fractionation kit (Cell Signaling Technology #9038, Danvers, MA, USA) according to the manufacturer’s protocol. Western blotting of cell fractions was performed using antibodies for NOXA (Thermo Fisher #MA1-41000, Waltham, MA, USA), MEK1 (BD Biosciences #610121), AIF (Cell Signaling #5318), and Histone H3 (Cell Signaling #4499) and analyzed by immunoblotting with ECL2 western blotting substrate (Thermo Fisher #32132). Images of the original blots are provided in Supplementary Figure S1.

### Annexin V staining and FACS analysis

MDA-MB-231 cells were seeded at 5.0 × 10^5^ cells in 60 mm plates in triplicate and were treated with either vehicle or Dox (1 µM) for 48 h. Following treatment, cells were trypsinized, collected, washed with PBS, and resuspended with 100 µL of 1 × Binding Buffer. Annexin-V-FITC (BioLegend #640945) and propidium iodide (PI) (Thermo Fisher #P3566) were added, and cells were incubated in a dark for 15 min at room temperature. Then, 400 µL of 1 × binding buffer were added to the suspension for analysis. Cells were analyzed using FACSCAN (BD FACSCanto) and using the BDFACSDiva software, and quantified the population as either double-negative, Annexin V-positive, PI-positive, or double-positive. In order to rule out false positivity of PI staining due to doxorubicin’s autofluorescence, we validated the results from this experiment using Annexin V- FITC and DAPI (instead of PI), which showed consistent results (data not shown).

### Sesnescence-associated β-galactosidase (SA-β-gal) staining

Histochemical SA-β-gal staining in MDA-MB-231 cells exposed to doxorubicin was conducted as described by Dimri et al.^32^, and as in our previous work^33–35^. Images were generated using bright field on an inverted microscope (Olympus inverted microscope IX70, 20 × objective, Q-Color3™ Olympus Camera; Olympus, Tokyo, Japan).

### Statistical analysis

Analyses of gene expression and IHC data were performed using GraphPad Prism version 9 (GraphPad Software, San Diego, California, USA). Shapiro–Wilk and Kolmogorov–Smirnov tests were used to test the normality of each dataset. Differences in normally distributed data were calculated using paired and/or unpaired t tests, while datasets that were not normally disturbed were analyzed using non-parametric tests such as Mann–Whitney and Wilcoxon signed rank tests to compare the differences between the pre- and post-NAC. Error bars show the median and range of each examined set. All reported *p* values were 2-tailed and *p* ≤ 0.05 was considered statistically significant.

## Results

### Evaluation of PAMIP1 expression in human breast tumor samples following the exposure to neoadjuvant chemotherapy (NAC)

**Figure 1:**
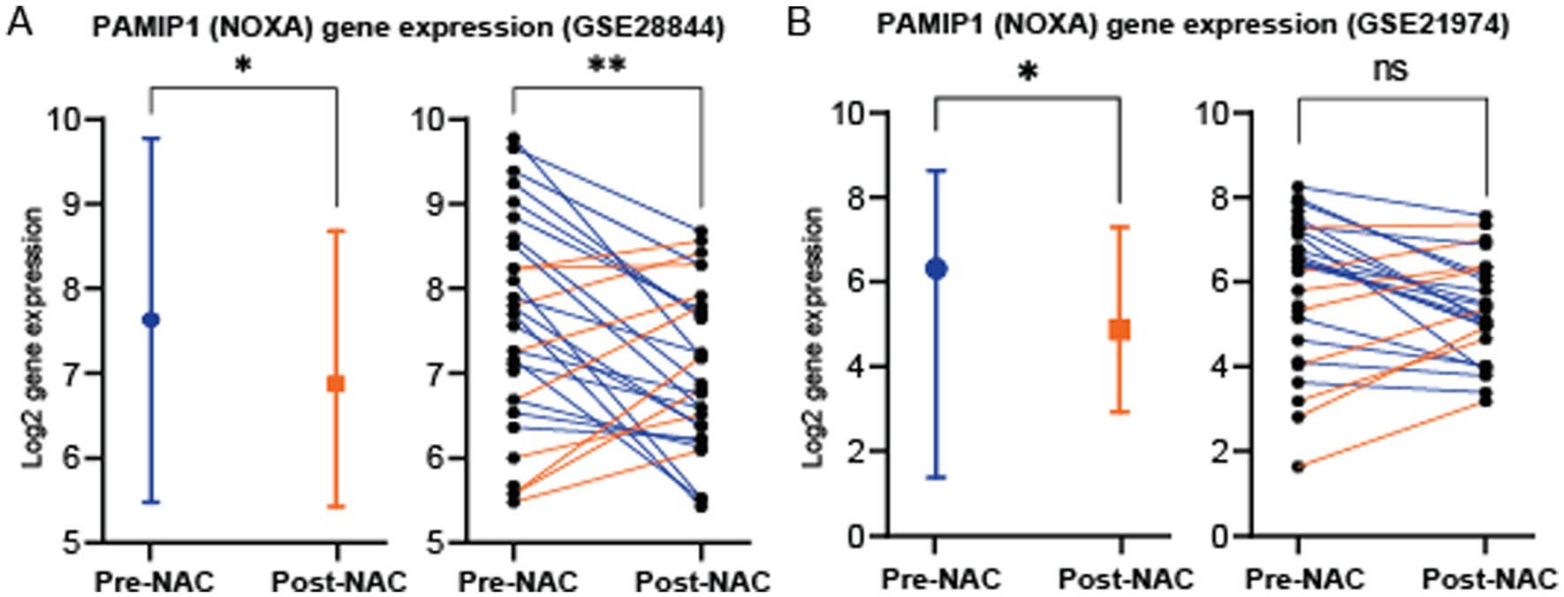
*PAMIP1* (NOXA) gene expression in two publicly available datasets of breast tumors that were exposed to NAC. (**A**) *PAMIP1* expression in a sample (GSE28844) of 33 invasive breast tumors before and after exposure to NAC (left panel). Of these, 28 were matched samples, collected before (pre-) and after (post-) NAC from each patient (right panel). In the matched set, 19 tumors showed reduced *PAMIP1* expression (blue lines), while only 9 showed increased expression (orange lines) following exposure to NAC. (**B**) *PAMIP1* expression in a sample (GSE21974) of 32 invasive breast tumors before (pre-) and after (post-) exposure to NAC (left panel), of which, 25 samples were matched (right panel). Similar to data in A, overall *PAMIP1* gene expression post-NAC was reduced (left panel), and 17 of the 25 matched samples exhibited a reduction in PAMIP1 expression (blue lines), and only 8 had increased expression (orange lines) following exposure to NAC. Statistical differences were calculated using unpaired and paired t-tests. ns: not statistically significant; *: *p* value < 0.05; **: *p* value < 0.01; ***: *p* value < 0.001; ****: *p* value < 0.0001. NAC: neoadjuvant chemotherapy.

We set out to investigate the effect of NAC on the expression of the NOXA gene, phorbol-12-myristate-13-acetate-induced protein 1 (*PAMIP1*), in breast cancer tissue. For that, two publicly available gene expression datasets of invasive breast cancer were investigated (GSE28844 and GSE21974) (Fig. 1)^30, 31^. Vera-Ramirez et al., collected a cohort of 33 breast cancer patients who received NAC as part of their treatment and whose both pre-NAC and post-NAC tumor samples were available for whole-genome expression analysis (GSE28844)^30^. Among this set (GSE28844), there was a statistically significant decrease in the *PAMIP1* gene expression following the exposure to NAC (*p* = 0.0466) (Fig. 1A, left panel). Of these 33 breast cancer patients, matched samples (pre- and post-NAC) were collected from 28 patients. Pairwise comparison of these 28 patients’ samples confirmed the downregulation in *PAMP1* expression following NAC as 19 matched tumor samples (68%) showed a decrease in *PAMIP1* gene expression (Fig. 1A, right panel), while only nine tumors (32%) showed increased expression (Fig. 1A, right panel) (*p* = 0.009).

Another cohort of 32 breast cancer patients that received NAC as part of their treatment was examined for the change in expression of *PAMIP1* (GSE21974)^31^. Similar to the previous set, there was a statistically significant decline in the *PAMIP1* gene expression following the exposure to NAC (*p* = 0.0307) (Fig. 1B, left panel). Among these 32 patients, there were 25 patients whose samples were collected pre- and post-NAC and the comparison of these 25 matched patient tumors showed that, similar to GSE28844 percentage, 68% (n = 17/25) of the samples had a decrease in the *PAMIP1* gene expression post-NAC (Fig. 1B, right panel), albeit was not statistically significant (*p* = 0.0637).

### Assessment of NOXA protein expression in human breast cancer samples in response to NAC

**Figure 2:**
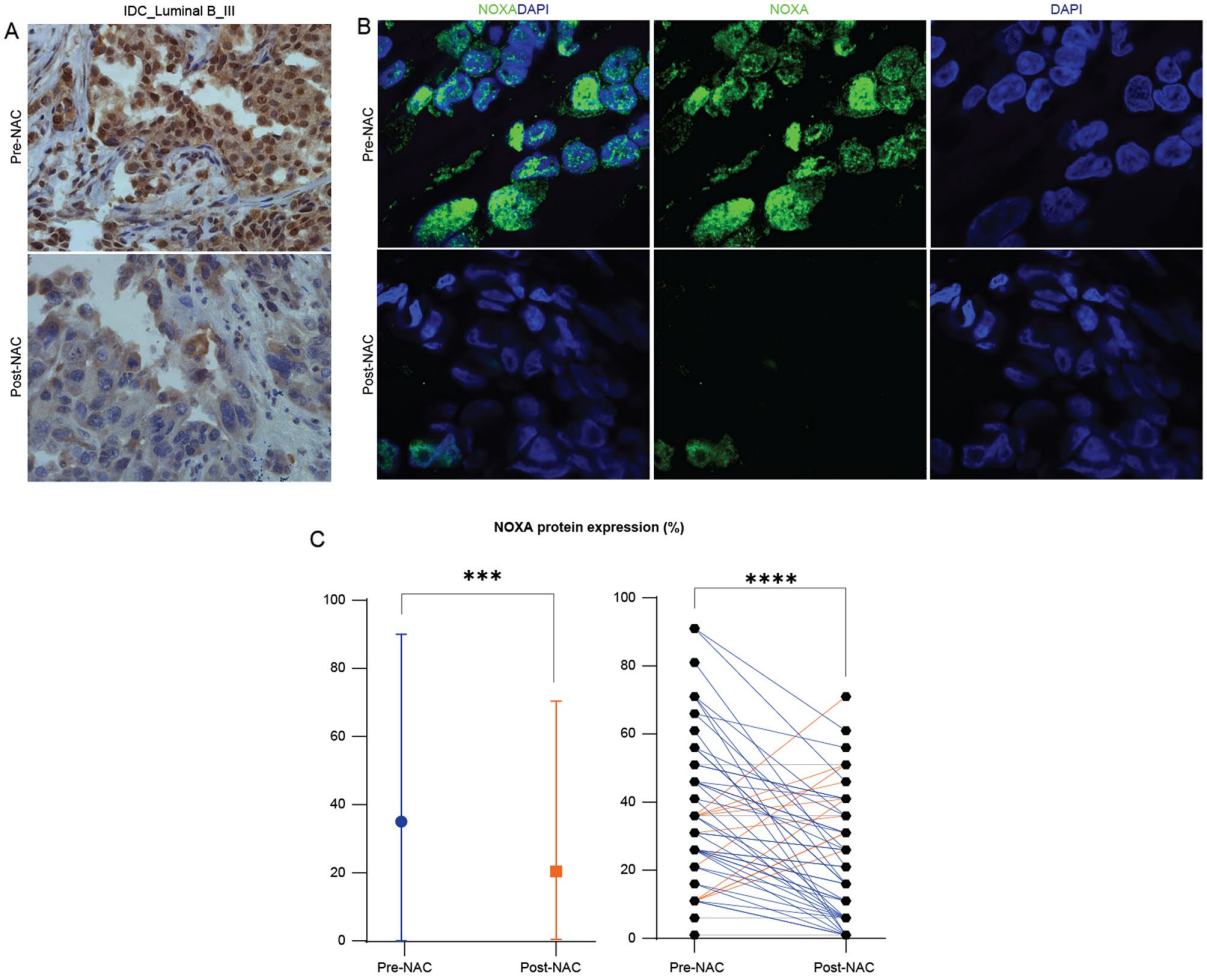
NOXA protein expression decreases post-NAC in invasive breast cancer samples. (**A**) Representative immunohistochemistry images of significant decrease in the overall expression of NOXA protein in a case of breast Invasive Ductal Carcinomas (IDC)**,** luminal B (HER2-\PR-\ER +), stage III. (**B**) Immunofluorescence (IF) images of NOXA protein in a case of breast IDC, luminal A (HER2-\PR + \ER +), stage I,  showing significant decline in NOXA overall expression post-NAC. DAPI was used as a nuclear marker. Images were acquired at × 100. (**C**) The overall decrease of NOXA protein expression in our 55 samples of invasive breast cancer that were collected pre- and post- NAC. Blue lines indicate the decrease in NOXA protein expression post-NAC, while orange lines indicate the increase in NOXA protein expression following NAC and gray lines indicate that no change in expression was recorded. Statistical differences were calculated using Mann–Whitney and Wilcoxon signed rank tests. Ns: not significant, *: *p* value < 0.05; **: *p* value < 0.01; ***: *p* value < 0.001; ****: *p* value < 0.0001. NAC: neoadjuvant chemotherapy.

We next investigated the NOXA protein expression in our cohort using immunohistochemical (IHC) techniques which was evident in breast cancer tissue (Fig. 2A,B). In a representative case of stage III IDC (luminal B), we observed a significant decline in the percentage of NOXA positively stained cells following exposure to NAC relative to pre-NAC (Fig. 2A). Furthermore, we performed immunofluorescence staining of NOXA protein, and the acquired confocal images confirmed the previous observations of downregulated expression of NOXA protein in the post-NAC samples compared to their matched pre-NAC (Fig. 2B). Next, we analyzed the overall IHC scores of NOXA protein expression in our cohort of 55 matched breast cancer patients that had received NAC as part of their management plan. The assessment of the overall NOXA protein expression levels revealed a statistically significant decline among the post-NAC samples relative to the pre-NAC set (Fig. 2C, left panel). Three quarters (75%) of the 55 matched pre- and post-NAC samples (n = 41/55) showed a decrease in levels of NOXA protein expression compared to just 18% (n = 10/55) which showed an increase in NOXA protein expression following NAC (Fig. 2C, right panel). In total, these findings were consistent with the gene expression analysis reflecting a significant decrease in NOXA levels as a response to NAC in breast tumors undergoing partial pathological response.

### Association of clinicopathological characteristics with the overall protein expression of NOXA in matched breast cancer samples

Next, we examined the association of the overall protein expression of NOXA with the clinicopathologic features of the 55-breast tumor patients. For this, we used the median of the pre-NAC as a cut-off point to dichotomize the expression into two groups: high (> 35%) and low (≤ 35%) positive staining of NOXA. There was a statistically significant correlation between higher protein expression levels of NOXA (> 35%) and increased patients’ age (> 49 years) among the pre-NAC (*p* = 0.0151) but not with post-NAC (*p* = 0.3911) samples (Table 2). Furthermore, we found that negative lymph node status (no tumor cells observed in lymph nodes) was correlated with increased expression levels of NOXA protein (*p* = 0.0266) (Table 2)*.* No other statistically significant associations were identified (Table 2).

**Table 2:** The correlation between NOXA protein expression and the clinicopathological features of the 55 patients’ sample.

		Pre-NAC		*P* value	Post-NAC		*P* value
		Low	High		Low	High	
Stage	1	11	14	0.9062	17	8	0.975
	2	8	8		11	5	
	3	7	7		10	4	
Age	≤ 49	18	10	**0.0151**	21	7	0.3911
	> 49	8	19		17	10	
LN status	Negative	6	16	**0.0266**	14	8	0.5571
	Positive	20	13		24	9	
Lymphovascular invasion	Negative	12	15	0.7891	19	8	> 0.9999
	Positive	14	14		19	9	
Grade	1	1	0	0.2352	1	0	0.7939
	2	14	11		17	8	
	3	11	18		20	9	
Type	IDC1	22	26	0.1236	34	14	0.08
	2ILC	3	0		3	0	
	3Other	1	3		1	3	
HER2	1Negative	17	19	> 0.9999	25	11	> 0.9999
	2Positive	9	10		13	6	
ER	1Negative	87	4	0.1988	9	2	0.4711
	2Positive	19	25		29	15	
PR	1Negative	9	9	> 0.9999	12	6	> 0.9999
	2Positive	17	20		26	11	
Luminal	Luminal A1	16	18	0.9992	23	11	0.6287
	Luminal B2	7	8		11	4	
	3HER2 +	2	2		2	2	
	TNBC	1	1		2	0	

NAC, neoadjuvant chemotherapy; IDC, Invasive ductal carcinoma; ILC, Invasive lobular carcinoma; LN, lymph node; ER, Estrogen receptor; PR, Progesterone receptor; HER2, Human epidermal growth factor receptor 2. Significant values are in bold.

### Establishing the association between therapy-induced senescence (TIS) and the protein expression of NOXA in human breast cancer samples

Finally, we investigated the association between the induction of TIS in response to NAC and the levels of NOXA protein expression among the matched sample sets that exhibited low levels of NOXA expression post-NAC. We selectively explored these samples because of the recent report by Shahbandi et al. showing that low levels of NOXA had a role in developing resistance for the BH3-mimetic, ABT-263, a widely known senolytic^28^. The induction of TIS as a response to NAC was identified based on the protein expression of three senescence-associated markers, namely, Lamin B1, Ki-67 and p16^INK4a^. Lamin B1, a structural component of the nuclear envelope^36^, is downregulated upon senescence induction^36, 37^. Since the identification of a single senescence-associated biomarker is insufficient to evaluate senescence^38^, we combined measuring Lamin B1 expression with the expression of p16^INK4a^ and Ki-67. p16^INK4a^ is a cell cycle regulator induced in response to DNA damage and cell stress and facilitates the senescence-associated growth arrest^39^. Previous studies have shown that the overexpression of p16^INK4a^ in breast cancer is an indicator of senescence induction^40^, specifically in TNBC and luminal B breast cancer^40, 41^. Lastly, the reduction of Ki-67 expression following exposure to NAC in breast cancer can be used as an indicator of the loss of proliferative capacity that characterizes TIS^42^. Accordingly, based on our paradigm, for a sample to be considered positive for TIS post-NAC, it must demonstrate a decline in Lamin B1 and Ki-67 along with an increase in p16^INK4a^ protein levels.

**Figure 3:**
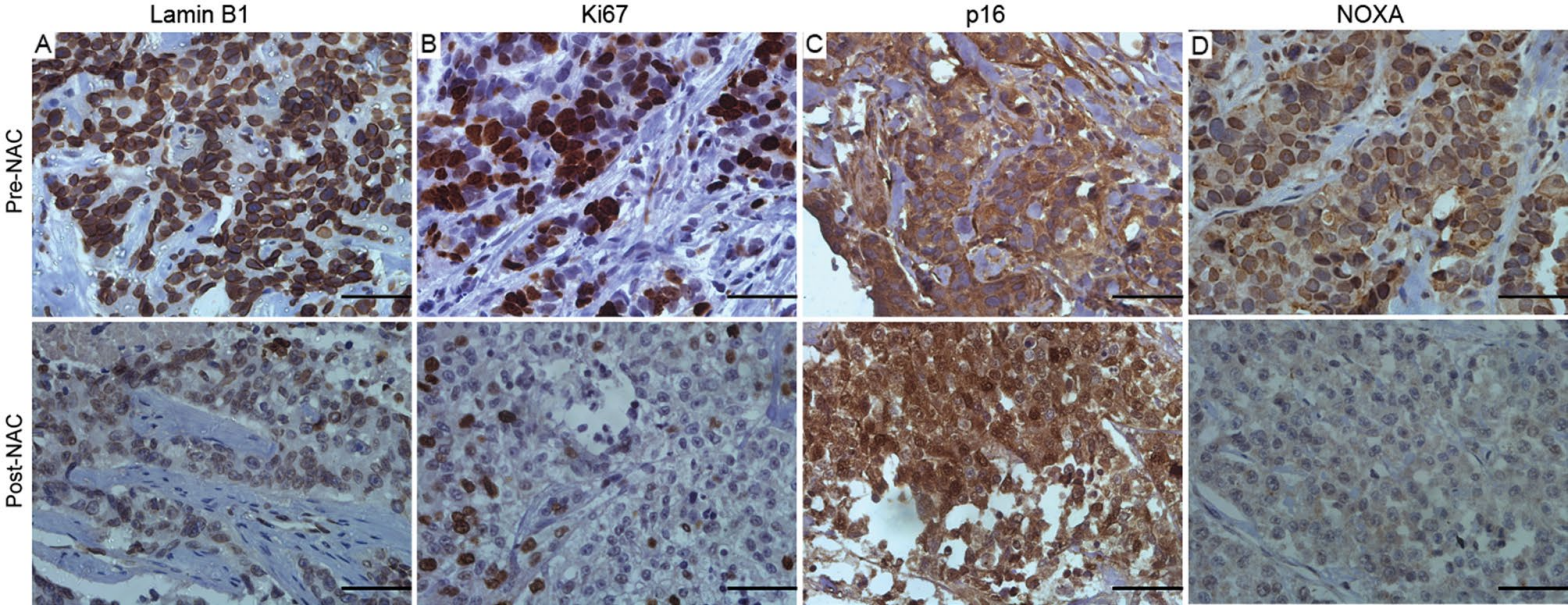
Representative fields of senescence-associated protein biomarkers and NOXA staining in invasive breast cancer. A representative case of stage II Invasive Ductal Carcinoma (IDC) of the breast before (upper panels) and after exposure to NAC (lower panels) stained by IHC for (**A**) Lamin B1, (**B**) Ki-67, (**C**) p16^INK4a^, and (**D**) NOXA. The figure shows the decline in Lamin B1 and Ki-67 with increase in p16^INK4a^; a signature indicative of TIS post-NAC accompanied with decreased NOXA protein expression**.** Cytoplasmic staining of p16^INK4a^ is considered negative staining^43^. Scale bars = 5 µm. Images were acquired at 40x.

We utilized the matched tumor samples in our cohort that exhibited a decline in NOXA protein expression post-NAC (n = 41) to investigate whether there is an association with the induction of TIS as determined by the decrease in Lamin B1 and Ki-67 accompanied by the increase of p16^INK4a^. Figure 3 shows a representative case of a breast cancer sample with decreased NOXA protein expression following NAC accompanied with possible evidence of TIS. Lamin B1 protein was remarkably downregulated following NAC (Fig. 3A, upper vs lower panels), and the same applied to the protein expression of Ki-67 (Fig. 3B, upper vs lower panels). On the other hand, an increase in the nuclear staining of p16^INK4a^ was observed under the same conditions (Fig. 3C, upper vs lower panels). Finally, NOXA protein expression was confirmed to be decreased after NAC as illustrated in Fig. 3D.

**Figure 4:**
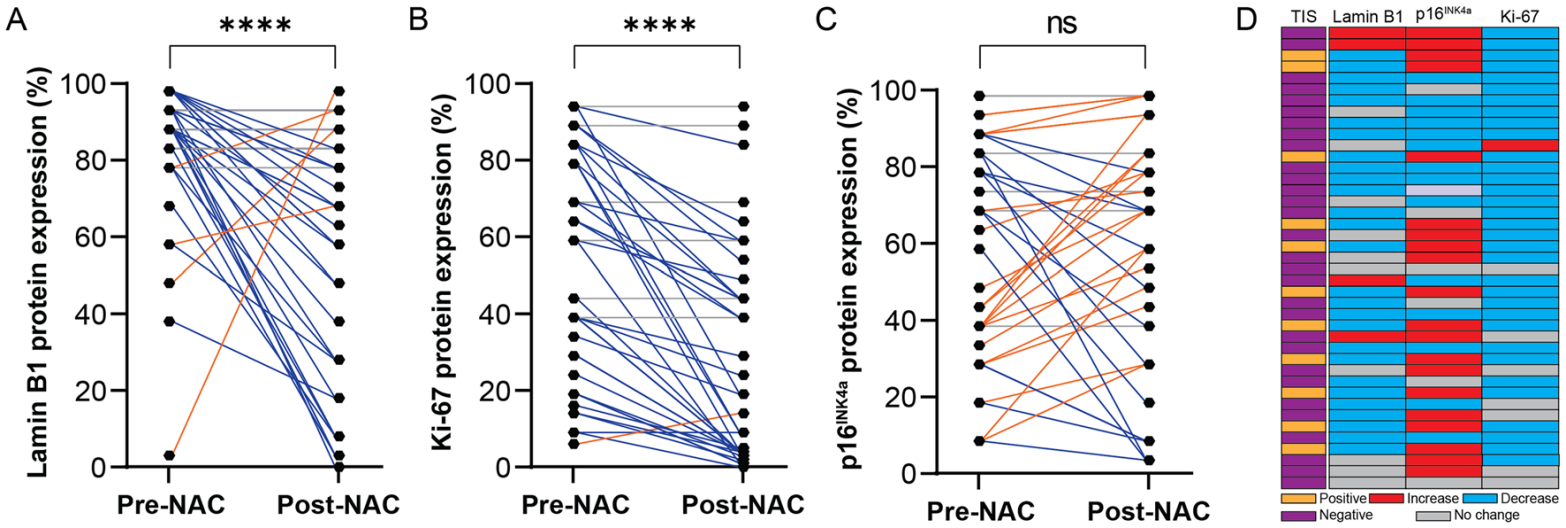
Analysis of the three senescence-associated biomarkers in a cohort of 41 matched samples that showed decreased NOXA protein expression post-NAC. Changes in protein expression based on IHC of (**A**) Lamin B1. (**B**) Ki-67. (**C**) p16^INK4a^ in each individual matched breast cancer sample set pre- and post-NAC. (**D**) Comparison of pre- vs. post- NAC for each patient for the three markers with possible positivity for TIS. Blue rectangles indicate a decrease in the senescence-associated protein expression post-NAC, while red rectangles indicate an increase in the senescence-associated protein expression following NAC, and gray rectangles indicate no change in expression was recorded. Statistical differences were calculated using Wilcoxon signed rank tests. ns: not significant, *: *p* value < 0.05; **: *p* value < 0.01; ***: *p* value < 0.001; ****: *p* value < 0.0001. NAC: neoadjuvant chemotherapy.

The analysis of TIS markers separately showed that the decline in Lamin B1 protein expression post-NAC was observed in 66% (n = 27/41) of the samples, while only 10% (n = 4/41) showed increase following NAC (*p* < 0.0001) (Fig. 4A). Moreover, the decline in Ki-67 protein expression was observed in 80% (33/41) of the samples compared to just one matched sample (2%) exhibited increase after NAC (*p* < 0.0001) (Fig. 4B). On the other hand, 49% (n = 20/41) of the samples showed an increase in p16^INK4a^ protein expression, whilst 34% (14/41) had declined in p16^INK4a^ protein expression following NAC intervention (*p* = 0.7446) (Fig. 4C). The final assessment of these 41 samples showed that 27% of the samples had a positive TIS profile (n = 11 patients) (27%) (Fig. 4D). Collectively, our analysis suggests that TIS represents a component of tumor biology in response to NAC in samples that fail to develop a pCR and that a subset of tumors with TIS are associated with downregulated NOXA expression.

### Assessment of NOXA protein expression in human breast cancer cells in vitro

**Figure 5:**
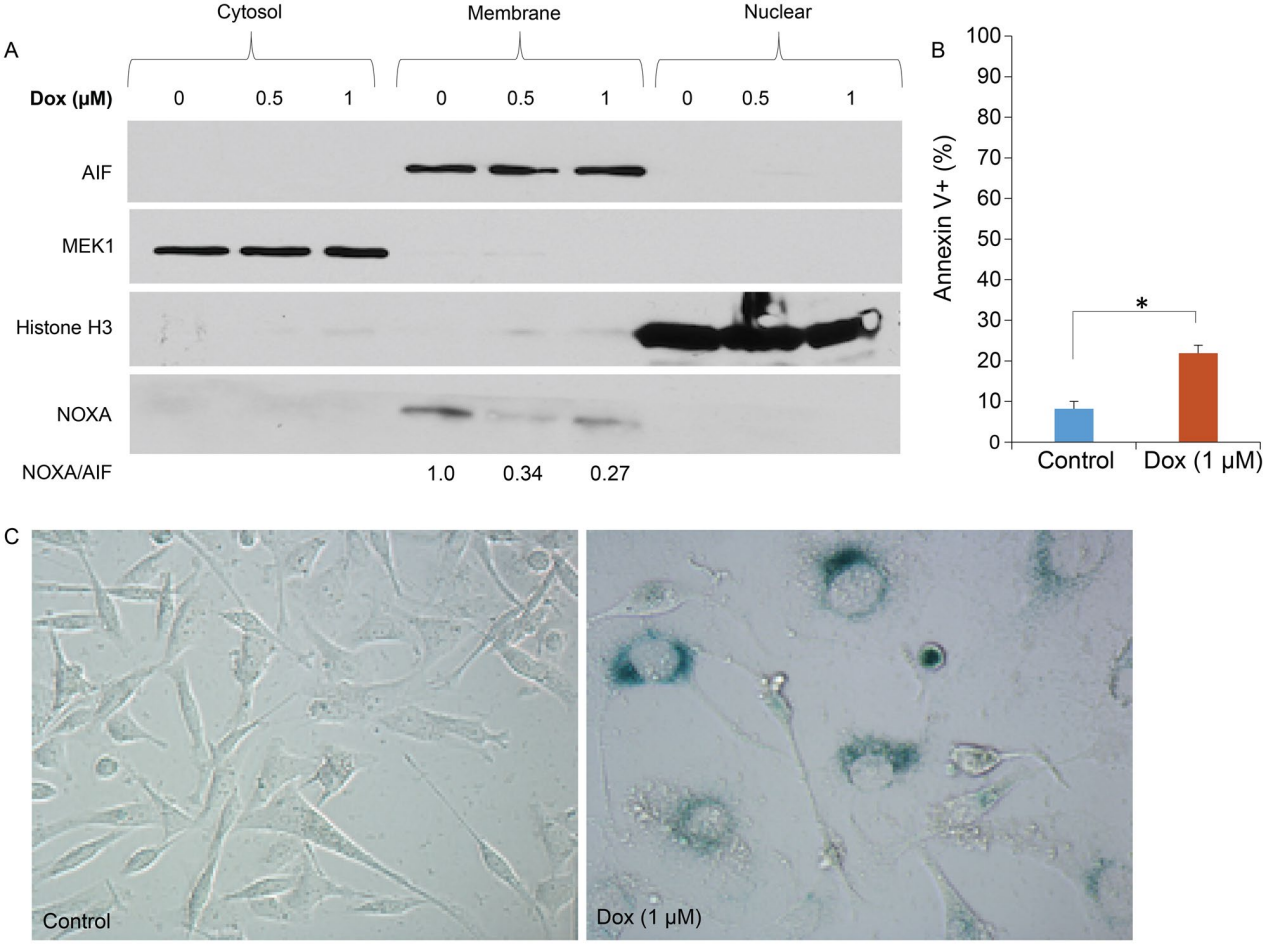
Downregulation of NOXA protein expression in response to doxorubicin in vitro. (**A**) MDA-MB-231 cells were treated with doxorubicin (Dox) for 48 h followed by cellular fractionation and Western blotting against NOXA, showing decreased expression of NOXA at the membrane with Dox treatment. Other protein markers were used as loading controls for each cellular compartment. Each protein was probed in a separate membrane then images were cropped and arranged in one figure to show relevant bands of corresponding proteins. Full images are shown in Supplementary Figure S1. (**B**) MDA-MB-231 cells were treated with vehicle or Dox (1 µM) for 48 h. Cells were harvested and treated with Annexin V-PI and FACS analysis was performed. *p < 0.05. (**C**) SA-β-gal staining of MDA-MB-231 cells exposed to Dox (1 µM) in comparison with their vehicle-treated counterparts. Both images were obtained at 200 × magnification.

In parallel, we confirmed these observations in a frequently utilized breast cancer cell line exposed to doxorubicin and investigated the changes in NOXA expression at different cellular compartments. NOXA expression was detected in the membrane fraction (mainly mitochondria) of MDA-MB-231 breast tumor cells (Fig. 5A and Supplementary Figure S1). Interestingly, NOXA protein expression was reduced following the exposure to increasing concentrations of doxorubicin (0.5 µM and 1 µM), which goes in agreement with our observations in the clinical breast cancer samples exposed to NAC (Fig. 5A). Moreover, MDA-MB-231 cells that showed downregulated NOXA expression in response to doxorubicin underwent minimal apoptosis (Fig. 5B), and were also positive for SA-β-gal upregulation (Fig. 5C), consistent with previous observations that breast tumor cells exhibit a senescent profile in response to chemotherapy^33^, and show reduced NOXA expression^28^.

## Discussion

Although tumor cell apoptosis is the desired outcome of cancer treatment including NAC, our limited understanding of the role of pro-apoptotic proteins, such as NOXA, in mediating tumor cell apoptosis in the clinical setting remains a challenge. Our study aimed at characterizing the protein expression of NOXA and its encoding gene *PAMIP1* following the exposure of breast cancer tissue to NAC. Moreover, we examined whether there was a relevant change in NOXA expression post-NAC in breast cancer samples exhibiting features of Therapy-Induced Senescence (TIS), a connection that is still poorly understood in clinical cancer. Particularly, it is still unknown which dictates the development of either apoptosis or TIS in response to chemotherapy, and whether the development of TIS instead of apoptosis explains the failure of breast cancer to develop pCR in response to chemotherapy^44^.

Our analysis of two publicly available datasets of breast cancer patients that were partly treated with NAC revealed a significant decrease of *PAMIP1* expression following the completion of NAC in both datasets that included pre- and post-NAC matched samples obtained from the same patient. The robustness of these observations is derived from the fact that these sets were obtained from different patients treated in different centers and receiving variable formulas of NAC, suggesting that the decline in *PAMIP1* expression post-NAC is a consistent observation. Subsequent immunohistochemistry staining and analysis of NOXA protein expression levels in our cohort of 55 breast cancer patients showed that three quarters of the samples (41/55) exhibited a downregulation in NOXA protein levels post-NAC confirming our findings of *PAMIP1* gene expression analyses. To our knowledge, this is the first report to investigate the NOXA protein expression levels in breast cancer tissue and its change following exposure to NAC.

Next, we utilized a senescence-detection signature of three senescence-associated biomarkers to determine the induction of TIS in the same samples^24^. Analysis of TIS markers in the 41 patient samples that showed downregulation in NOXA protein expression revealed a decline of Lamin B1 and Ki-67 in most of the samples at 66% and 80%, respectively, and an upregulation of p16^INK4a^ in 49% of the samples. Combined analysis of these markers showed that 27% (n = 11) of the 41 NOXA-downregulated cohort had a profile suggestive of TIS, indicating that there is a possible correlation between the failure to undergo pCR and the development of TIS and/or low NOXA.

Few studies investigated *PAMIP1* gene expression or NOXA protein expression in clinical cancer samples, and as to our knowledge, the study by Karbon et al., is the only one conducted in breast cancer^15^. In this study, the authors found higher *PAMIP1* mRNA expression in 92 frozen breast cancer tissue samples relative to 10 non-tumor breast samples and that increased expression of NOXA/*PAMIP1* was found to be associated with improved relapse-free survival (RFS) and overall survival (OS). The authors extended their analysis to investigating a 1,060-case breast cancer TCGA dataset of which 44% of patients received chemotherapy, confirming that high NOXA/*PAMIP1* predicts favorable therapy outcome^15^. These findings are commensurate with our hypothesis that functional NOXA is important in the execution of successful apoptosis in response to therapy, and that a decline in NOXA expression following therapy is likely to be associated with suboptimal responses to chemotherapy^15, 28^. In other cancers, Kosmidou et al. reported that *NOXA* mRNA was overexpressed in 66% of the examined colorectal cancer tumor samples relative to their paired non-malignant colonic tissue counterparts^45^. However, the authors performed IHC staining for NOXA protein in 39 samples that “showed high levels of differential mRNA expression”^45^, but found negative NOXA expression in some cases and did not report detailed findings on the IHC staining, suggesting that the IHC approach might be a better assay than mRNA expression for NOXA detection. Unfortunately, further evidence on the evaluation of NOXA protein expression levels in clinical cancer samples is parsimonious.

The identification of senescence in human tissue samples is challenging, largely due to the variability in the expression of the frequently utilized senescence-associated biomarkers between in vitro and in vivo models^46^. However, it remains essential to investigate these markers to generate evidence on the nature and extent of senescence development in the clinical setting. This is of particular importance since the induction of senescence in cancer and non-cancer cells in response to the exposure to various forms of antitumor therapy has been recently linked to deleterious outcomes of therapy. In breast cancer, DNA damaging, cytotoxic drugs were shown to trigger TIS based on SA-β-gal staining, p53 and p16^INK4a^ expression in 36 breast cancer samples from patients who received neoadjuvant chemotherapy^7^. Of those 36 samples, 15 (41%) samples stained positively for SA-β-gal. In a similar work, we have looked into the expression of three senescence-associated proteins, namely, p21^Cip1^, Lamin B1, and H3K9Me3 in 37 breast cancer tissue samples exposed to NAC and developing partial or incomplete pathological response^26^. We specifically considered samples that are positive for p21^Cip1^, positive for H3K9Me3 and negative for Lamin B1 to be reflective of TIS induction following exposure to chemotherapy, in an attempt to utilize the co-existence of multiple senescence hallmarks within the same tumor tissue to more rigorously identify TIS^22^. Like results from Poele et al.^7^, TIS was identified in 40.54% of breast cancer samples exposed to chemotherapy. However, we noticed that markers such as p21^Cip1^ and H3K9Me3 are less likely to be reliable indicators for TIS in breast cancer since their protein expression levels do not undergo significant changes after exposure to chemotherapy, while Lamin B1 undergoes significant reduction and potentially provides a better in vivo marker of TIS^47^.

The inconsistency in some of the established markers to reflect a senescence status in vivo has been documented previously. For example, Domen et al. used a signature of lipofuscin accumulation, high p16^INK4a^ and p21^Cip1^ expression, and low Ki-67 staining to identify TIS in lung cancer samples^48^. While being able to demonstrate evidence of senescence in lung cancer tissue overall, the three markers, p21^Cip1^, p16^INK4a^ and Ki-67, did not show different expression levels when measured in samples obtained from patients that did not receive chemotherapy^48^. Furthermore, the existence of the holistic senescence signature based on the four markers combined was also not significantly different when compared between chemotherapy- and non-chemotherapy-treated patients, indicating that this work lacked firm evidence of TIS^48^. Accordingly, in this work, we utilized three senescence markers that somewhat consistently suggested a TIS status, i.e., in samples with significant Lamin B1 reduction following NAC exposure, levels of Ki-67 were most likely to be reduced, and p16^INK4a^ to be increased. Nevertheless, it is quite important to stress that no combination of biomarkers have been rigorously validated to measure TIS in breast cancer tissue, or any other type of cancer for that matter, and a more thorough marker analysis of in vivo transcriptomic and proteomic signatures is required.

What determines whether apoptosis or senescence is induced (or a combination of) in clinical cancer following exposure to chemotherapy is not fully understood. One hypothesis is that when tumors develop pCR they are likely to have undergone apoptosis as the major response to NAC^49–51^. Alternatively, failure to undergo an adequate tumor shrinkage could be explained by the development of apoptosis resistance and/or the induction of other cell stress responses such as TIS which allows, in part, for the persistence of tumor cells^52^. Unfortunately, one limitation in our study is that we could not investigate the changes in NOXA and TIS-associated signature in the post-NAC, post-mastectomy samples undergoing pCR, simply because those samples cannot be collected. However, our analysis revealed that a substantial portion of breast tumors that failed to undergo pCR display markers of TIS and downregulate NOXA, hinting that both these processes might be implicated in mediating therapy resistance. In fact, Shahbandi et al. showed that a fraction of senescent breast tumor cells resists cell death through downregulation of NOXA in vitro^28^, which is confirmed in this work in breast cancer samples exhibiting a senescence-like profile. Moreover, studies by Shahbandi et al. showed that the low NOXA expression in senescent tumor cells confers resistance to senolytics, and thus, represents a major barrier against their use as adjuvant cancer treatment to eliminate residual senescent tumor cells^28^. Our work provided clinical evidence that supports this concern and strongly invites further characterization of the susceptibility of senescent tumor cells to senolytics before they can be translated to the clinic. Subsequently, it is not unlikely that a fraction of tumor cells undergoes TIS and downregulate NOXA in the clinical setting which not only interfere with the development of pCR, but also might exhibit resistance against the potential use of certain senolytics. More careful screening of cancer patients is required prior to the utilization of senolytics in cancer therapy to achieve optimal individualized therapy.

### Supplementary Information

Supplementary Figure S1.

## Data Availability

The patients’ datasets generated during the current work are not publicly available due to patients’ privacy concerns of the institutional review board policies on human tissue data but are available upon request from the corresponding author. The data of the gene expression profiles that were utilized in support the findings of this work are available from the Gene Expression Omnibus (GEO) database with reference numbers: GSE28844 (https://doi.org/10.1371/journal.pone.0053983) and GSE21974 (https://doi.org/10.3892/or.2011.1392).
