# Supplementary material for: NOXA expression is downregulated in human breast cancer undergoing incomplete pathological response and senescence after neoadjuvant chemotherapy
Source: Sci Rep. 2023 Sep 23;13:15903. doi: 10.1038/s41598-023-42994-2 (PMC10517932; doi:10.1038/s41598-023-42994-2)
Supplement: Supplementary file 1 — Supplementary Figure S1. [file 41598_2023_42994_MOESM1_ESM.docx]

**Supplemental Information**

NOXA expression is downregulated in human breast cancer undergoing incomplete pathological response and senescence after neoadjuvant chemotherapy

Sofian Al Shboul, Mohammed El-Sadoni, Ahmad Alhesa, Nisreen Abu Shahin, Dua Abuquteish, Ola Abu Al Karsaneh, Elham Alsharaiah, Mohammad A. Ismail, Liliya Tyutyunyk-Massey, Moureq R. Alotaibi, Victoria Neely, Hisashi Harada, Tareq Saleh


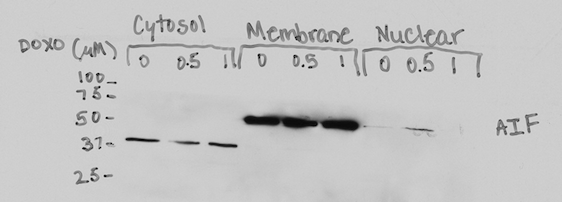


AIF (67 kD)

A


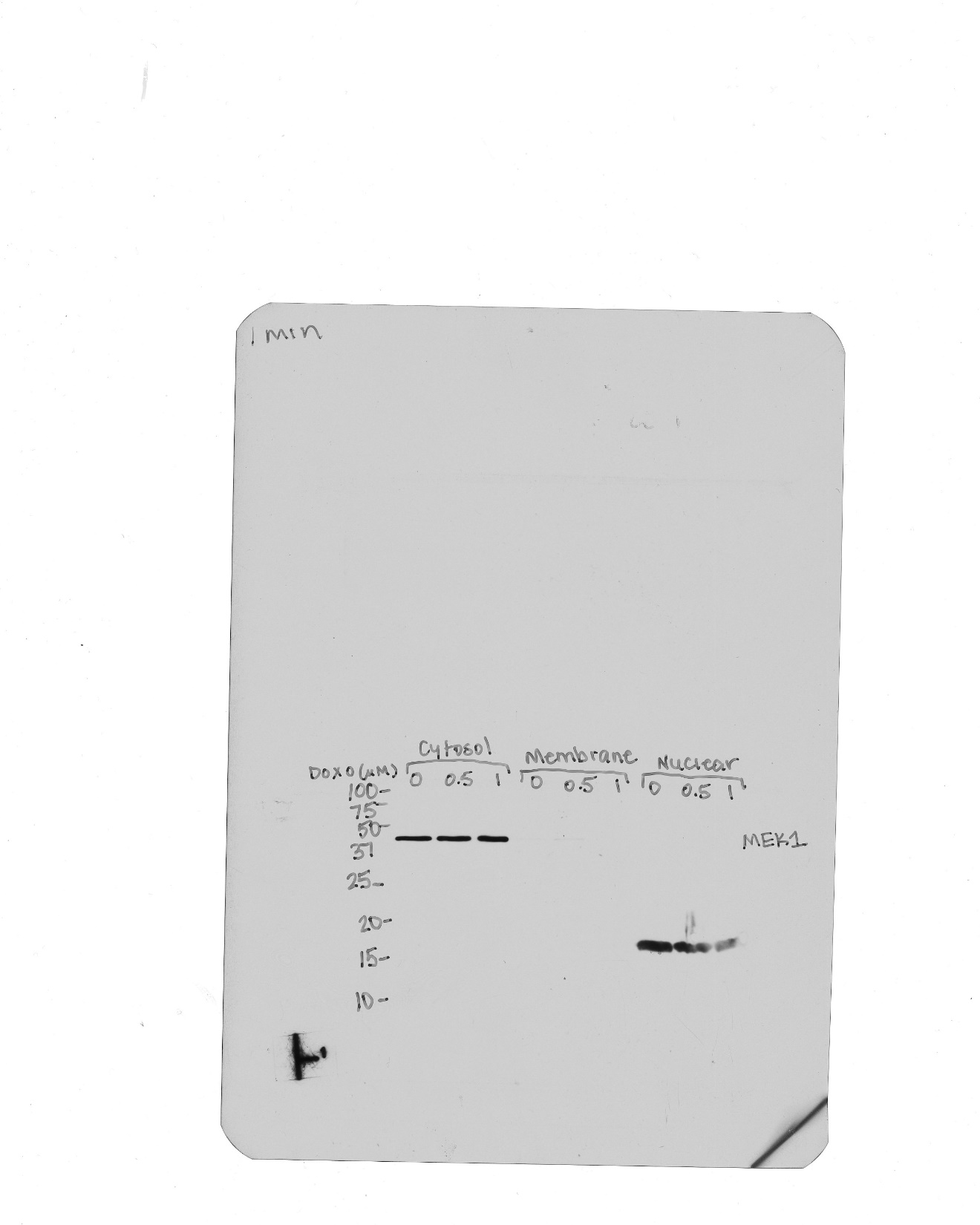


MEK1 (45 kD)

B


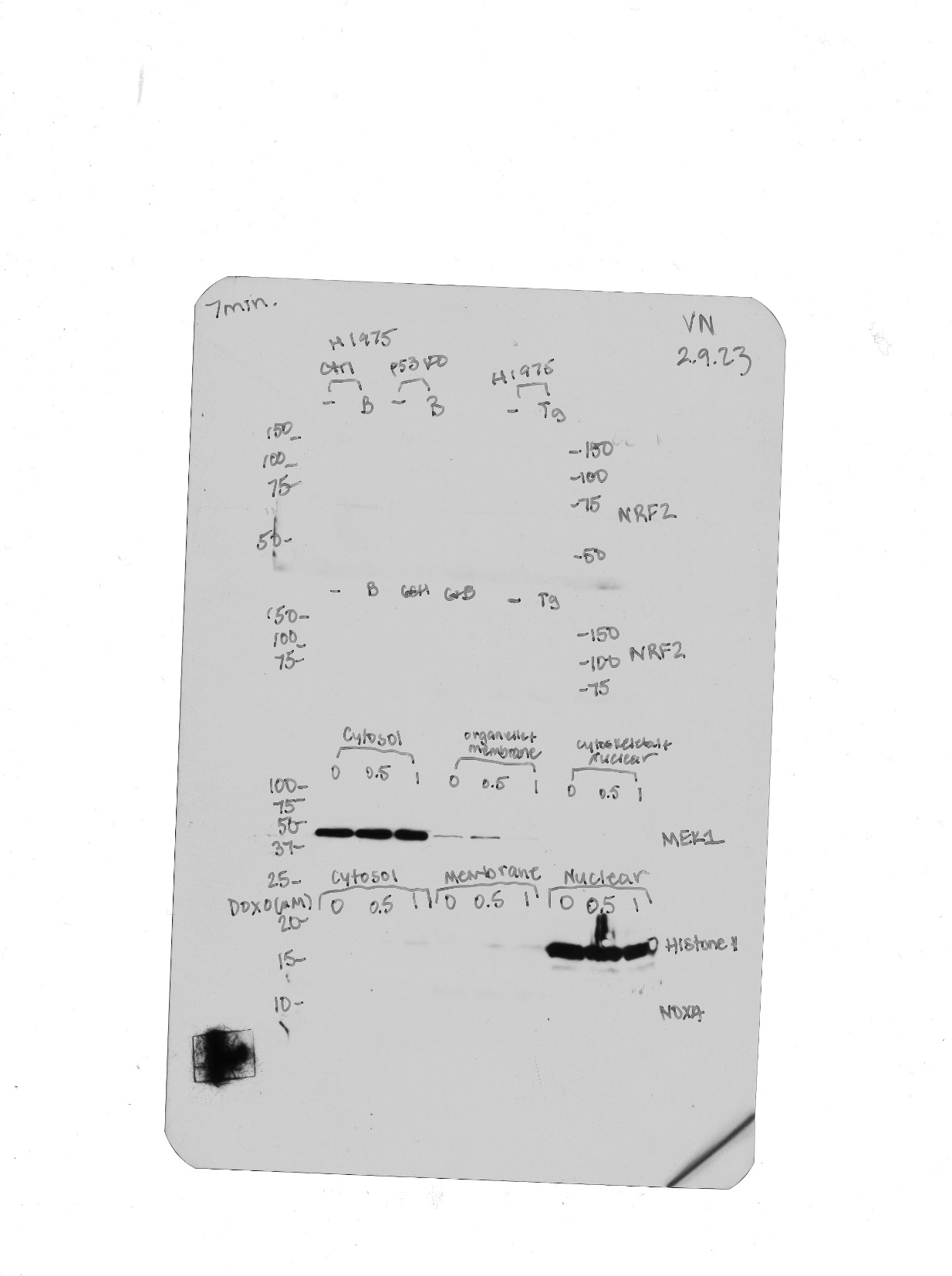


Histone H3 (17 kD)

C


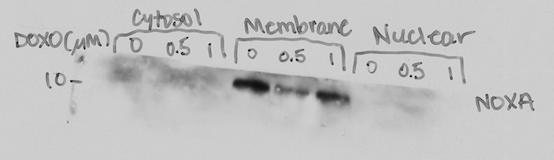


NOXA (10kD)

D

**Supplementary Figure S1: Uncropped original blots showing the decrease of NOXA protein expression following exposure to doxorubicin (DOX) for 48h in MDA-MB-231 cells.** Cellular fractionation and immunoblotting against **A.** AIF, **B.** MEK1, **C.** Histone H3 and **D.** NOXA. Subcellular fractionation was conducted using the Cell Fractionation kit (Cell Signaling Technology #9038, Danvers, MA, USA) according to the manufacturer’s protocol. Western blotting of cell fractions was performed using antibodies for NOXA (Thermo Fisher #MA1-41000, Waltham, MA, USA), MEK1 (BD Biosciences #610121), AIF (Cell Signaling #5318), and Histone H3 (Cell Signaling #4499). Blots were sliced prior to hybridization with antibodies. The 45 kD bands in cytosol in **A** are the remining of the MEK1 bands since this blot was re-probed with AIF following the MEK1 antibody.
